# Benthic species of the Kerguelen Plateau show contrasting distribution shifts in response to environmental changes

**DOI:** 10.1002/ece3.4091

**Published:** 2018-05-24

**Authors:** Charlène Guillaumot, Salomé Fabri‐Ruiz, Alexis Martin, Marc Eléaume, Bruno Danis, Jean‐Pierre Féral, Thomas Saucède

**Affiliations:** ^1^ Marine Biology Lab CP160/15 Université Libre de Bruxelles (ULB) Brussels Belgium; ^2^ UMR CNRS 6282 Biogéosciences Université de Bourgogne Franche‐Comté (UBFC) Dijon France; ^3^ Département Adaptation du Vivant Museum National d'Histoire Naturelle UMR BOREA 7208 Paris France; ^4^ Département Origine et Évolution Museum National d'Histoire Naturelle UMR ISYEB 7205 Paris France; ^5^ Aix Marseille Université CNRS UMR 7263 IMBE Marseille France

**Keywords:** conservation, echinoid, future predictions, historical changes, Kerguelen Plateau, species distribution modeling

## Abstract

Marine life of the Southern Ocean has been facing environmental changes and the direct impact of human activities during the past decades. Benthic communities have particularly been affected by such changes although we only slowly understand the effect of environmental changes on species physiology, biogeography, and distribution. Species distribution models (SDM) can help explore species geographic responses to main environmental changes. In this work, we modeled the distribution of four echinoid species with contrasting ecological niches. Models developed for [2005–2012] were projected to different time periods, and the magnitude of distribution range shifts was assessed for recent‐past conditions [1955–1974] and for the future, under scenario RCP 8.5 for [2050–2099]. Our results suggest that species distribution shifts are expected to be more important in a near future compared to the past. The geographic response of species may vary between poleward shift, latitudinal reduction, and local extinction. Species with broad ecological niches and not limited by biogeographic barriers would be the least affected by environmental changes, in contrast to endemic species, restricted to coastal areas, which are predicted to be more sensitive.

## 1 INTRODUCTION

The consequences of global climate change in the polar seas are predicted to lead to warmer, fresher, and more acidic waters, in addition to more extreme climatic events and seasonal variations than actual conditions (Allan et al., 2013; Gutt et al., 2015). Significant changes have already been recorded in Antarctic waters; for instance, sea surface water temperature in the western Antarctic Peninsula has increased by 1°C over the last half‐century (Turner et al., 2013). All these changes are critical for Antarctic organisms as they lead to a decrease in habitat suitability for the species (Clarke et al., 2007; Doney et al., 2011). Sub‐Antarctic ecosystems are confronted with the direct and indirect impacts of climate change too (i.e., glacier retreat, temperature increase, decrease in precipitations), with the combined effects of these multiple stressors also leading to the prevalence of favorable climatic conditions for introduced species and, consequently, to alterations in the pristine marine life (Allan et al., 2013; Byrne, Gall, Wolfe, & Agüera, 2016; Kargel et al., 2014; Molinos et al., 2015; Pendlebury & Barnes‐Keoghan, 2007; Smith, 2002).

Physiological responses of marine species to changes in seawater temperature, salinity, and more acidic conditions have been assessed by ecophysiological experiments (Collard, De Ridder, David, Dehairs, & Dubois, 2015; Karelitz et al., 2016; Peck, Souster, & Clark, 2013; Suckling et al., 2015), which are usually confronted with practical limitations due to substantial technical and funding issues (Suckling et al., 2015). Species geographic responses to the multiple effects of climate change may include resilience, distribution range shift toward the pole, where they would find more suitable conditions, and local extinction (Doney et al., 2011; Walther et al., 2002). Species distribution models (SDM) have been currently used to address these biogeographic issues for conservation purposes (Marshall, Glegg, & Howell, 2014; Reiss et al., 2014; Ross & Howell, 2013; Zucchetta, Venier, Taji, Mangin, & Pastres, 2016), assess the direct impact of human activities on ecosystems (Vázquez‐Luis, March, Álvarez, Álvarez‐Berastegui, & Deudero, 2014; Vierod, Guinotte, & Davies, 2014), and predict species distribution range shifts in response to climate change (Guillera‐Arroita et al., 2015; Tingley, Vallinoto, Sequeira, & Kearney, 2014). SDM relate species occurrence records to abiotic environmental predictors (Elith & Leathwick, 2009; Elith et al., 2006) to identify species suitable areas (Phillips et al., 2017). Species distribution range shifts can be modeled by projecting species suitable areas in geography using key predictive environmental descriptors and different climate scenarios for the near future (Guisan & Thuiller, 2005; Reiss et al., 2014).

Applied SDM studies to marine species (Duffy & Chown, 2017; Marshall et al., 2014; Robinson et al., 2011; Ross & Howell, 2013) may be also confronted with substantial limitations. Sampling bias, data availability, quality, and heterogeneous distribution are common issues (Guillera‐Arroita et al., 2015; Robinson et al., 2011; Tessarolo, Rangel, Araújo, & Hortal, 2014). However, protocols have been developed to address these methodological issues and provide robust and relevant distribution predictions (Barbet‐Massin, Jiguet, Albert, & Thuiller, 2012; Guillaumot, Martin, Eléaume, & Saucède, in press; Phillips et al., 2009).

In the Southern Ocean, echinoids are common components of marine benthic communities. Nearly 200 species with contrasting ecological niches (David, Choné, Mooi, & de Ridder, 2005) were recorded (Fabri‐Ruiz, Saucède, Danis, & David, 2017). They also constitute substantial elements of trophic networks (Marina et al., 2016; Raymond et al., 2011), can promote benthic diversity (Linse, Walker, & Barnes, 2008; Pabis, Sicinski, & Krymarys, 2011), and play a key role in benthic ecosystems (Brey & Gutt, 1991; David et al., 2005). For instance, cidaroids were listed as vulnerable marine ecosystem (VME) indicator taxa by CCAMLR (Convention for the Conservation of Antarctic Marine Living Resources, CVD code) because of their rich epibiont assemblages (Hardy, David, Rigaud, De Ridder, & Saucède, 2011; Linse et al., 2008), brooding behavior, lack of motility, and sensitivity to fishing activities (CCAMLR 2009).

In this work, SDM were used to predict the geographic response to environmental changes of four common echinoid species of the Kerguelen Plateau with contrasting ecological niches: *Abatus cordatus*,* Brisaster antarcticus*,* Ctenocidaris nutrix,* and *Sterechinus diadema*. Recent observations indicate that the Kerguelen Plateau is a highly dynamic region currently undergoing significant ecological changes in response to climate warming (Allan et al., 2013; Byrne et al., 2016; Molinos et al., 2015). The objectives were to test whether changing environmental conditions might really impact local species distribution range in a near future, a critical issue for conservation strategies. To address this issue, SDM were generated for each species separately and for three different environmental conditions belonging to different time periods: past conditions [1955–1974], present‐day situation [2005–2012], and near‐future predictions [2055–2099].

## 2 MATERIALS AND METHODS

### 2.1 Studied area

The Kerguelen Plateau is a vast and remote area of the Southern Ocean that displays unique oceanographic features and proximity between marine fronts generating strong latitudinal temperature and salinity gradients (Moore & Abbott, 2002; Park et al., 2014). Important micronutrient releases, including iron, and high chlorophyll a concentrations are present on the eastern margin of the plateau. This contrasts with the “High Nutrient Low Chlorophyll” waters reported in most of the Southern Ocean (Koubbi et al., 2016; Park et al., 2014). High diversity levels in pelagic and benthic ecosystems are also described on the Kerguelen Plateau in comparison with the surrounding oceanic areas (Féral et al., 2016; Koubbi et al., 2016). The Kerguelen Plateau makes part of the French and Australian protected areas and aggregates substantial conservation issues for marine biodiversity due to fast environmental changes and the impact of fisheries activities (CCAMLR 2008, Koubbi et al., 2016; Welsford, Constable, & Nowara, 2011; Welsford, Ewing, Constable, Hibberd, & Kilpatrick, 2014). It was proposed that the recorded climate changes may correspond to a southward shift of the Antarctic Circumpolar Current [ACC] and of its frontal systems, in particular the sub‐Antarctic Front [SAF] and the Polar Front [PF] (Allan et al., 2013). This makes marine biodiversity of the region particularly at risk with regard to environmental changes. Alteration in marine biodiversity and ecosystem functioning is particularly expected to impact coastal marine areas of the Kerguelen Islands (CCAMLR 2008, 2013, Hureau, 2011). The relative low and direct anthropogenic impacts on the Kerguelen marine ecosystems make this archipelago a relevant sentinel to assess the direct effects of actual environmental changes on sub‐Antarctic marine habitats. In this context, the National Nature Reserve of the French Southern Territories was recently extended to most of the French Exclusive Economic Zone around the Kerguelen Islands over around 400,000 km^2^ (decree issued on 12 December 2016) following the commitments of France to the COP21 meeting held in Paris in 2015 (http://www.gouvernement.fr/action/la-cop-21, accessed on 29 September 2017). This makes the reserve the sixth world's largest Marine Protected Area (https://gis.ccamlr.org/home, accessed on 29 September 2017).

### 2.2 Biological records

**Figure 1:**
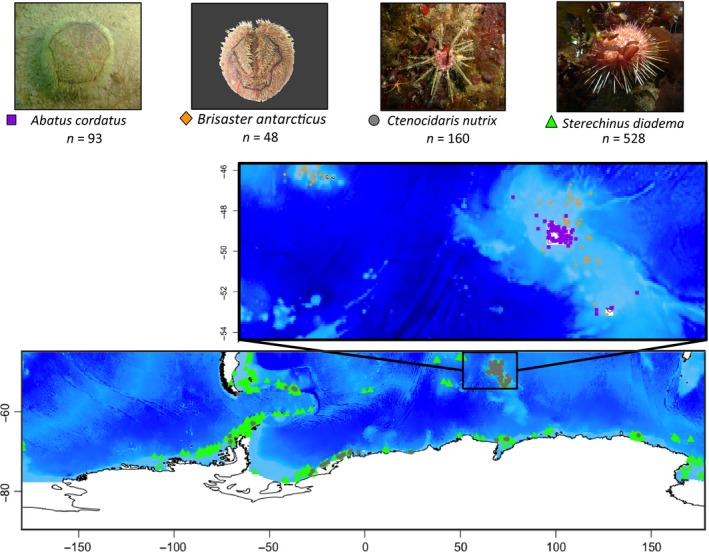
Distribution of presence‐only records available for the four studied species (occurrence duplicates were removed)

Echinoid species records were obtained from Fabri‐Ruiz et al. (2017). The dataset contains presence‐only data of 201 echinoid species collected in the Southern Ocean from the Antarctic coasts to 45°S latitude. This dataset is a compilation of data collected during oceanographic campaigns undertaken between 1872 and 2015. Four species with contrasting ecological requirements were selected in the dataset for this study. The four selected species are known by a sufficient number of presence records to perform robust species distribution models. These four species are common on the Kerguelen Plateau and constitute substantial representatives of Antarctic benthic ecosystems (De Ridder, David, & Larrain, 1992; Díaz, Féral, David, Saucède, & Poulin, 2011; Hardy et al., 2011; Linse et al., 2008; Moya, Saucède, & Manjón‐Cabeza, 2012; Poulin & Féral, 1995). Namely, we selected *Abatus cordatus* and *Brisaster antarcticus*, two species endemic to sub‐Antarctic regions, and *Ctenocidaris nutrix* and *Sterechinus diadema* that present broader distribution ranges in the Southern Ocean (Figure 1). Duplicate records that fell on one single grid‐cell pixel of environment layers were removed from the dataset.

### 2.3 Environmental data

The environmental descriptors used in this study were generated and described by Fabri‐Ruiz et al. (2017). They are available as raster layers collected from different sources and modified to fulfill modeling requirements at the scale of the Southern Ocean. Environmental data cover the extent of the Southern Ocean (<45°S) at a grid‐cell resolution of 0.1 degrees (around 10 km). Neighbor‐joining interpolation was applied to correct for missing values that may interfere with certain distribution modeling approaches. The dataset contains environmental descriptors for the six decades included between 1955 and 2012. Environmental data were processed using the functions proposed by the R (R Core Team 2015) *SDMPlay* (Guillaumot, Martin, Eléaume, & Saucède, 2016) and *raster* packages (Hijmans 2016).

Predictor selection is a major concern that can alter modeling performances (Braunisch et al., 2013; Petitpierre, Broennimann, Kueffer, Daehler, & Guisan, 2017). Here, we tested for collinearity between predictors and removed one descriptor from the initial dataset for VIF > 5 (variance inflation factor with the stepwise procedure of the *usdm* R package (Naimi, Hamm, Groen, Skidmore, & Toxopeus, 2014)) and Spearman correlation >0.85 (Dormann et al., 2013). A total of 15 descriptors were selected to run the models (Table S1).

Environmental descriptors were declined for three time periods: [1955–1974], [2005–2012], and the future projection of the IPCC scenario RCP 8.5 (IPCC Fifth Report, 2013) for [2050–2099]. Oxygen concentration and POC export layers were not available for future projections. Similarly, chlorophyll a, oxygen concentration, POC export, and ice coverage layers were not available for past projections. Therefore, these environmental parameters were considered constant and similar to the present values (Table S1).

### 2.4 Distribution modeling

Species distributions were modeled using the boosted regression trees (BRT) modeling technique (Breiman, 2001). BRT was shown to present high stability and transferability performances for limited and biased datasets (Reiss, Cunze, König, Neumann, & Kröncke, 2011; Heikkinen, Marmion, & Luoto, 2012; Guo et al., 2015; Guillaumot et al. in press.). BRT was calibrated according to Elith, Leathwick, and Hastie (2008). The combination of parameters that minimizes the predictive deviance of the models (learning rate lr, tree complexity tc, and bag fraction bf) were set at, respectively, lr = 0.0001, tc = 2, and bf = 0.75. Modeling computation was performed using the *gbm* R package (Ridgeway, 2015).

Presence‐only methods imply using background data to be selected in the study area. In the Southern Ocean, sampling effort is spatially contrasted and such spatial heterogeneities can alter modeling performances (Araújo & Guisan, 2006). We corrected for spatial bias using a target‐background sampling method (Phillips et al., 2009). The kernel density estimation (KDE) of visited pixels was estimated (i.e., grid cells in which at least one echinoid is recorded, according to the database compiled by Fabri‐Ruiz et al. (2017)). KDE layer is a proxy for the survey effort that is used to spatially weight background sampling and compensates for the weight of frequently visited sites in the models. As suggested by Barbet‐Massin et al. (2012), we selected a number of background records similar to the number of presence‐only records available and applied a 100‐fold replication of the background sampling in each case.

To take into account the limitations of model extrapolation, modeling areas were limited in geography and depth. On the basis of species records and ecology (David et al., 2005), we considered 1,000 m depth as a maximal extrapolation threshold for *A. cordatus* and *S. diadema,* and 1,500 m depth for *B. antarcticus* and *C. nutrix*. Boundaries in latitude and longitude were species‐specific and determined according to the most extreme positions of species records.

The area under the receiver operating characteristic curve (AUC) was used to evaluate models' robustness. AUC is an unbiased metric (i.e., threshold independence) that is unaffected by low‐prevalence datasets, which are typical in presence‐only data samples (Fielding & Bell, 1997; Hand, 2009; Proosdij, Sosef, Wieringa, & Raes, 2016).

Distribution models were generated for the present time period defined as decade [2005–2012] using environmental descriptors available for decade [2005–2012] and presence‐only records collected between 1974 and 2015, considering the minimum number of records needed to build robust models. This was considered an acceptable trade‐off as main temperature and salinity shifts occurred before 1974 in Antarctic and sub‐Antarctic regions (Frenot, Gloaguen, Picot, Bougère, & Benjamin, 1993; Meredith & King, 2005; Whitehouse et al., 2008). Based on these models, projections were made for the historical period covering the couple of decades [1955–1974] and for the predicted future period (RCP 8.5, [2050–2099]), using the respective environmental descriptors available for the two periods (Table S1). To assess the magnitude of environmental shifts that occurred between the three time periods, the significance of environmental shifts was tested between the environmental layers used for modeling using a Wilcoxon paired test.

### 2.5 Species distribution changes

Three indices were used to analyze model outputs and compare distribution maps between periods. They were adapted from Crase et al., (2015) (Table 1) to (1) quantify the extent of the distribution area as the sum of the presence probabilities (*E*_occupied_), (2) compare the relative raw difference between distribution maps indicating the overall change in the distribution of presence probabilities (*E*_instability_), and (3) analyze the relative overlap between distribution maps indicating any change in the distribution of suitable areas (*E*_overlap_). To compare index values between species, *E*_instability_ and *E*_overlap_ were scaled by *E*_occupied_ [2005–2012] providing possible scores included between 0 and 1 for the two indices.

**Table 1:** Indices adapted from Crase et al. (2015) for our case study

	Formula	Description
*E*_occupied_	$\sum_{i=1}^{n}p_{i}$	Sum of presence probabilities
*E*_instability_	$\sum_{i=1}^{n}\left|q_{i}-p_{i}\right|/\sum_{i=1}^{n}p_{i}$ [2005–2012]	Relative raw difference between distribution maps indicating the overall change in the distribution of presence probabilities
*E*_overlap_	$\sum_{i=1}^{n}p_{i}\cdot q_{i}/\sum_{i=1}^{n}p_{i}$ [2005–2012]	Relative overlap between distribution maps indicating any change in the distribution of suitable areas

*i* corresponds to a grid‐cell pixel, and *p* and *q* are the species distribution maps produced for the three time periods ([1955–1974], [2005–2012], and [2050–2099]). *E*_instability_: High values indicate important overall changes in the distribution between periods. *E*_overlap_: High values indicate similar highly suitable areas between periods.

In each SDM, we used the MaxSSS index (maximum sensitivity plus specificity) as threshold value between suitable and nonsuitable areas and turned raw distribution maps into red (suitable) and yellow (nonsuitable) binary maps. The MaxSSS was suggested to be the best index for presence‐only datasets (Liu, White, & Newell, 2013). It was measured using the *dismo* R package (Hijmans et al. 2016). Species distribution maps and changes between periods were analyzed on species‐specific extents, the four species being recorded on the Kerguelen Plateau.

To analyze the magnitude of environmental shifts that occurred between periods, the environmental subspace modeled as suitable for each species was estimated on the extent of the Kerguelen Plateau only (−56° to −46°S, +63° to +81°E). The total environmental space was defined as the total set of values present on the Kerguelen Plateau for the parameters that most contribute to the models. The portion of space occupied by each species (i.e., the occupied environmental subspace or species realized niche) was delineated by convex hulls and positioned based on the convex hull centroids using the *SIBER* R package (Jackson et al., 2011). Convex hulls correspond to polygons that delineate threshold values and comprise each species suitable environment. Size and position of convex hulls were compared between the three time periods.

## 3 RESULTS

### 3.1 Environmental changes through time

Environmental differences were tested between the three time periods of reference, for the eight environmental descriptors available for each period specifically (Table S1). Differences in the mean and amplitude of seafloor salinity and temperature were tested for significance (Wilcoxon test, *p* < .05) between [1955–1974], [2005–2012], and [2055–2099] according to scenario RCP 8.5. Differences in chlorophyll a values were also tested for significance between [2005–2012] and [2055–2099] (Wilcoxon test, *p* < .05). These results indicate that significant environmental changes have occurred in the recent past and might happen in a near future, potentially affecting species geographic range.

### 3.2 Environmental contributions and the spatial scale

**Figure 2:**
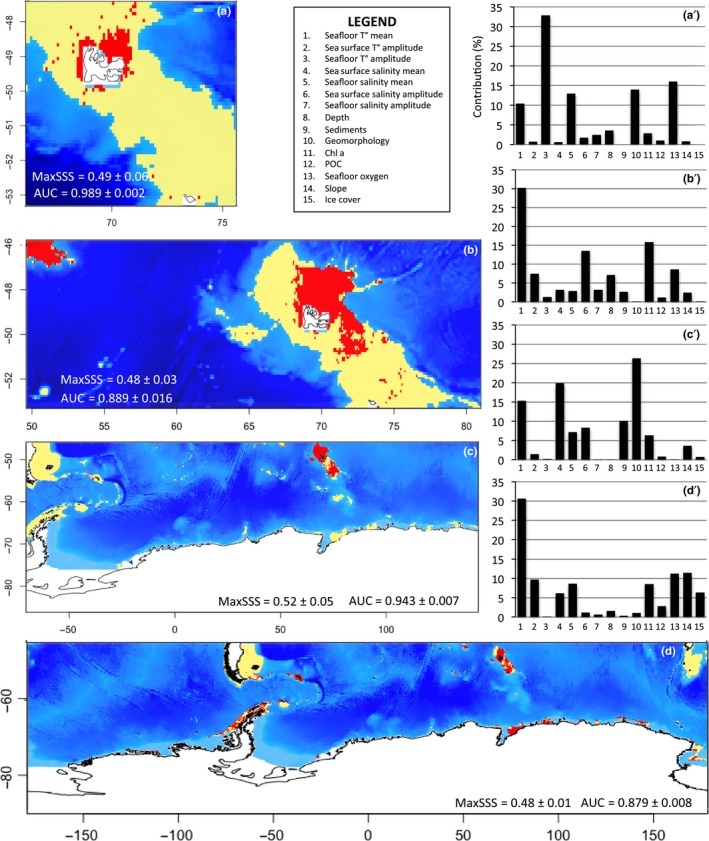
Binary plots of modeled species distribution on [2005–2012]. Yellow pixels: suitable area; red pixels: nonsuitable area. The map size is representative of the modeling boundaries for each species. (a) *Abatus cordatus*, (b) *Brisaster antarcticus*, (c) *Ctenocidaris nutrix*, and (d) *Sterechinus diadema*; (a'–d') contribution (%) of environmental descriptors provided by the [2005–2012] model for the four species on their respective modeled extent

All SDM generated for the present period [2005–2012] show high AUC values (AUC > 0.879; Figure 2), indicating high modeling relevance. The modeled species distributions show different distribution patterns, also contrasting in the extent of the predicted suitable area (Figure 2a–d) and the contribution of the environmental descriptors to each model (Figure 2a'–d'). The extent of suitable areas ranges from the regional scale to the entire Southern Ocean. The distribution of *A. cordatus* is exclusively predicted in coastal shallow waters (depth <200 m) of the Kerguelen Islands (Figure 2a). In contrast, the distribution of *S. diadema* is modeled from the sub‐Antarctic area to the Antarctic coasts (Figure 2d).

Contributions of environmental parameters differ between species models and are dependent on the extent of species distribution range. This is to be related to the geographic scale of descriptor variations. chlorophyll a values vary at the local scale, with high concentrations in certain areas of the Kerguelen Plateau and Crozet Islands. Coastal waters are also characterized by high temperature and salinity amplitudes in contrast to offshore areas (Figure S1). At broader scale, contrasts between descriptor values are less marked, but latitudinal gradients are noticeable in sea ice concentration and temperature values between Antarctic and sub‐Antarctic regions. The SDM produced for *B. antarcticus* shows a high contribution of chlorophyll a concentrations, mean seafloor temperatures, and mean sea surface salinities (Figure 2b'). The predicted distribution of this deposit‐feeder is obviously determined by the occurrence of high chlorophyll a blooms during the austral summer on the Kerguelen Plateau and in Crozet Islands (Figure 2b; Figure S1). In contrast, *C. nutrix* is more widely distributed (Figures 1 and 2c) and high contributions to the models are represented by environmental descriptors that vary at broader scale, such as geomorphology, mean sea surface salinities, and mean seafloor temperatures (Figure 2c'). The same holds true for the widely distributed *S. diadema* with a high contribution of mean seafloor temperatures (30.6%) to the SDM (Figure 2d').

### 3.3 Species modeled ecological niches

**Figure 3:**
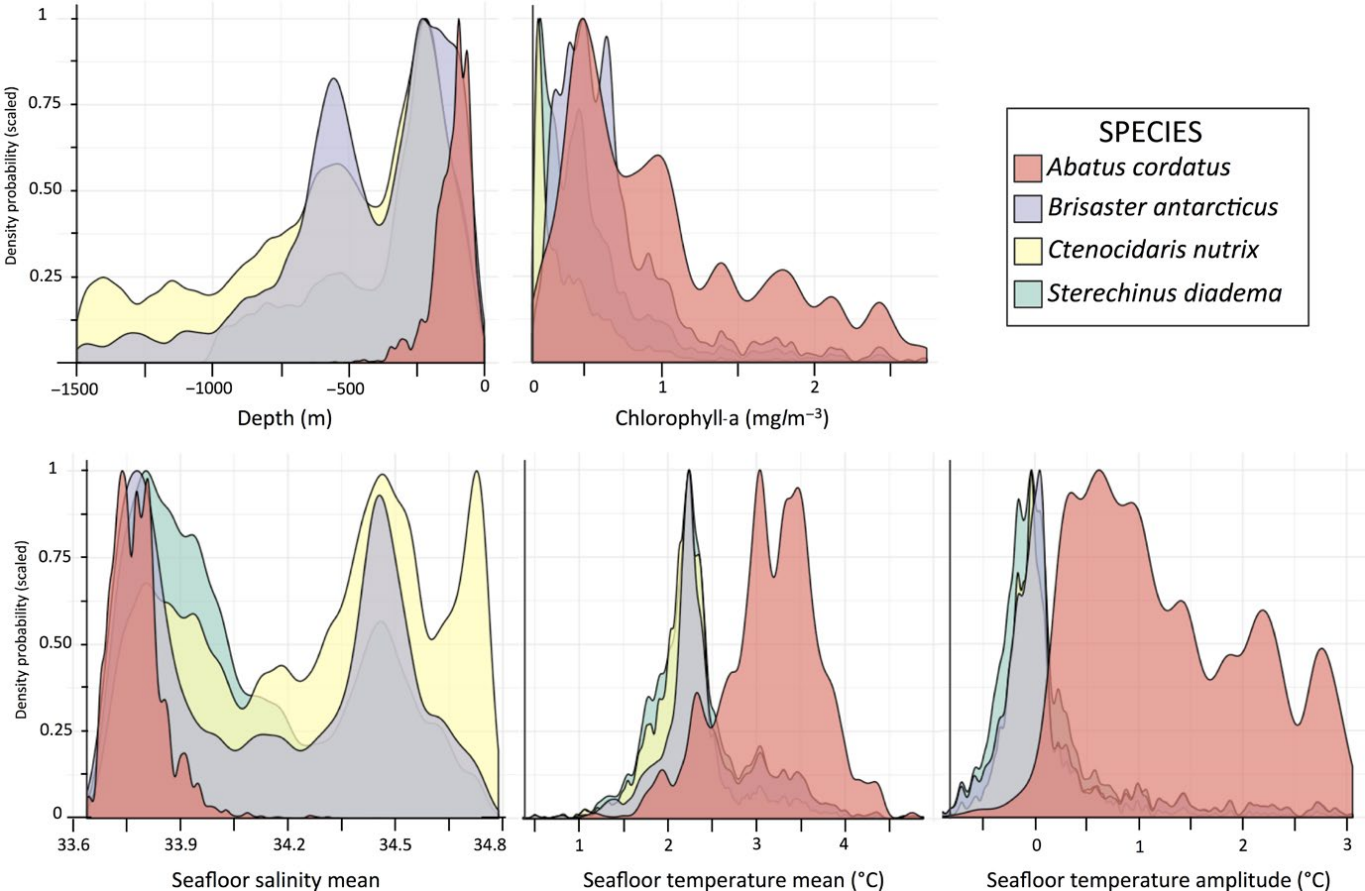
Scaled density probability of environmental occupied space, modeled as suitable for the four species on the extent of the Kerguelen Plateau (−46° to −56°S, +63° to +81°E). Suitability is defined by the MaxSSS threshold

The modeled environmental spaces occupied by the four studied species indicate contrasting realized niches. Results show that *A. cordatus* is a shallow‐water species (Figure 3) endemic to coastal waters of the Kerguelen Islands with suitable areas characterized by high temperature amplitudes, low salinity values (33.6 to 33.9 PSU), and temperatures contained between 3 and 4°C (Figure 3; Figure S2). Environmental preferences are quite narrow and suitable habitats restricted to a small area. Environmental preferences of the three other species are wider, although *B. antarcticus* distribution is restricted to the Kerguelen and Crozet archipelagoes (Figures 1 and 3). *B. antarcticus* is deposit‐feeder and shows preferences for areas with high chlorophyll a enriched waters. The species shows a heterogeneous depth distribution that can be explained by the recurrence of a deep plateau and slopes in the area (Figure S1). *C. nutrix* and *S. diadema* have both a wide distribution range extending from sub‐Antarctic to Antarctic regions and wide ecological niches (Figure 1). Mean seafloor salinity values are bimodal for *C. nutrix*, which is distributed in coastal waters of low salinity values in the Kerguelen Islands (33.7 to 34 PSU) as well as in offshore areas, in waters of high salinity values (Figure 3; Figure S1). Temperature tolerance of *S. diadema* is high (−1°C to +5°C) with preferences for low temperatures on the extent of the Southern Ocean (Figure S2).

### 3.4 Species distribution range shifts

**Figure 4:**
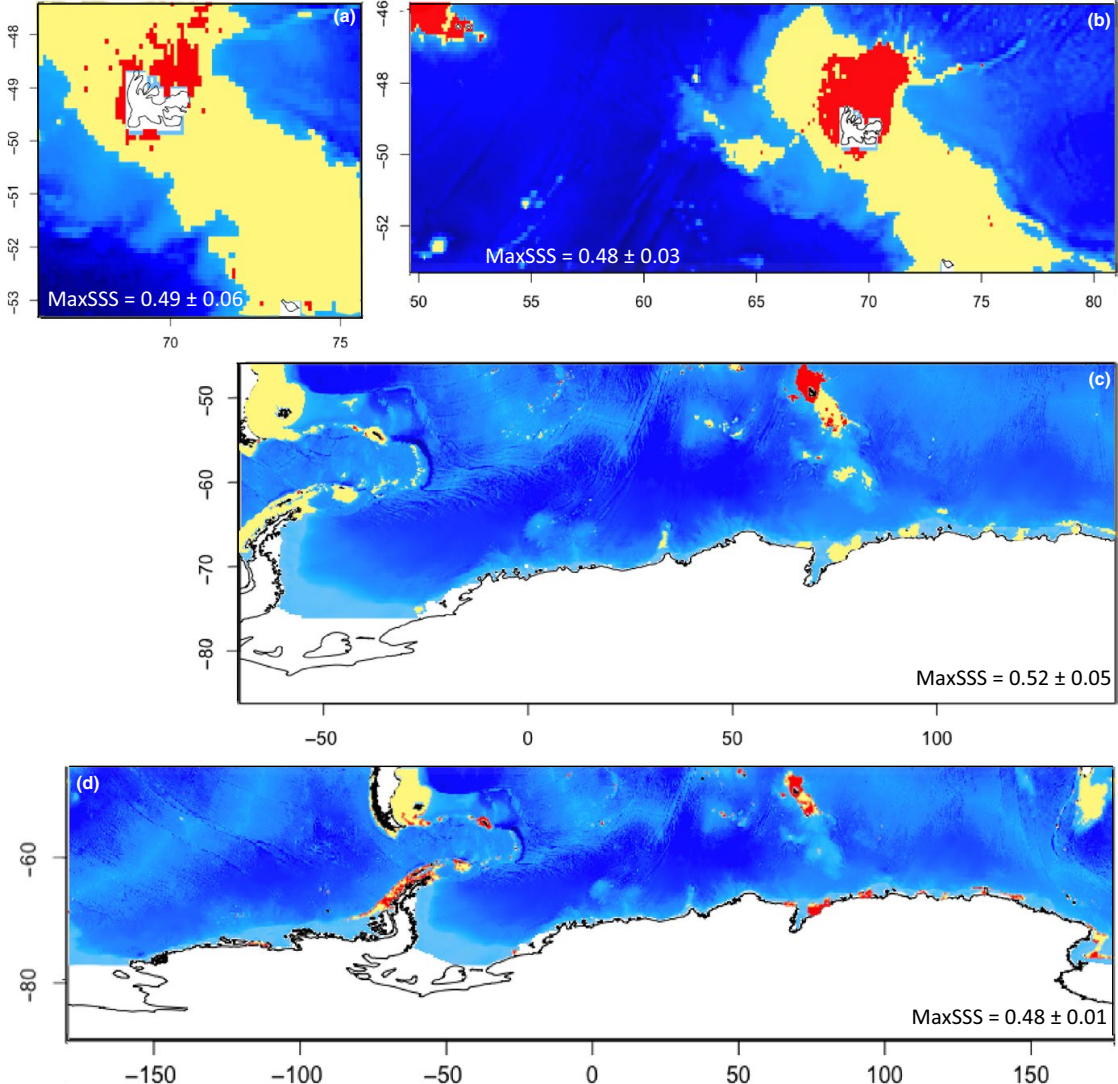
Binary plots of modeled species distribution projected on [1955–1974]. Yellow pixels: suitable area; red pixels: nonsuitable area. The map size is representative of the modeling boundaries for each species. (a) *Abatus cordatus*, (b) *Brisaster antarcticus*, (c) *Ctenocidaris nutrix*, and (d) *Sterechinus diadema*

**Figure 5:**
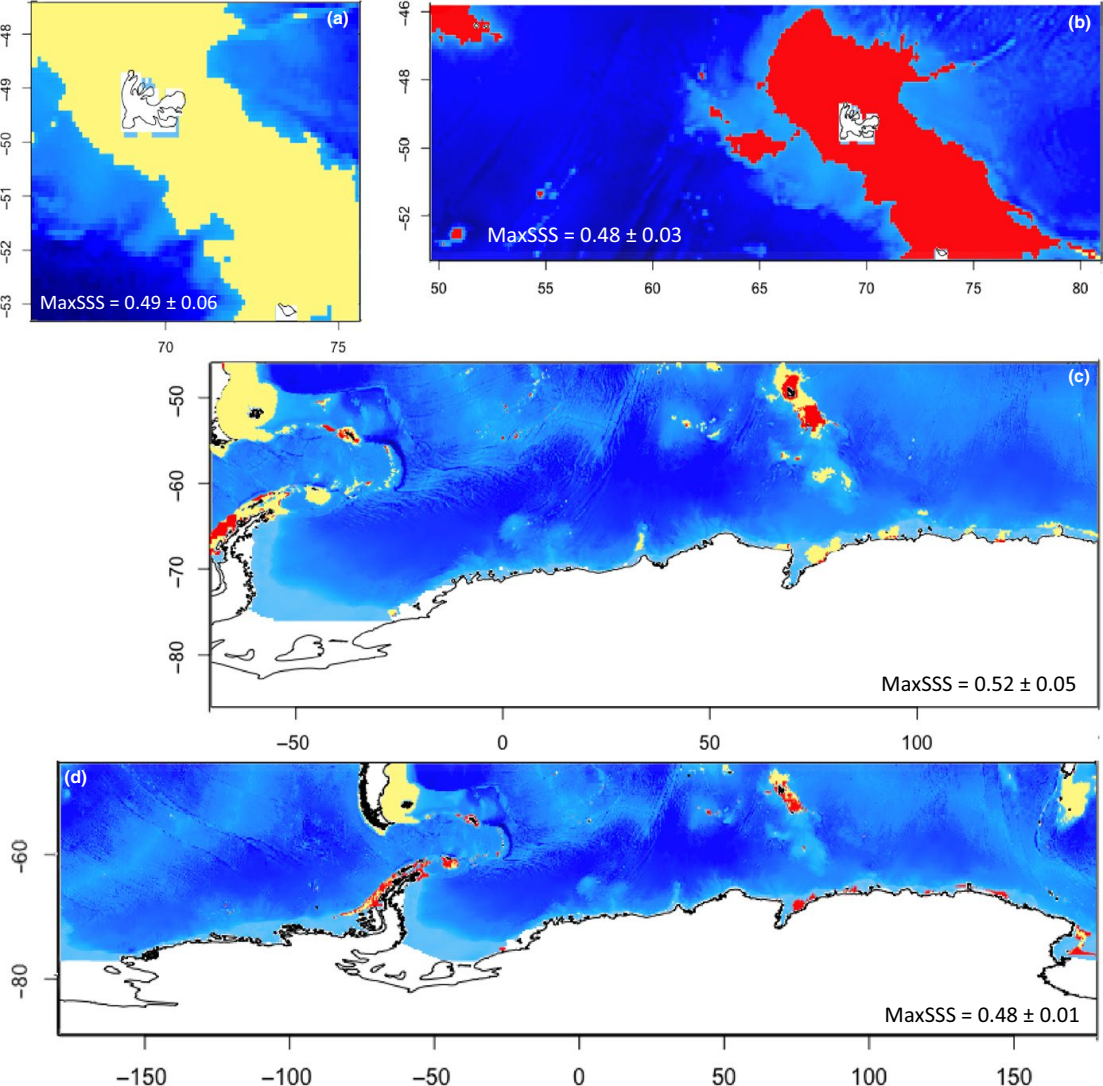
Binary plots of modeled species distribution projected on [2050–2099], scenario RCP 8.5. Yellow pixels: suitable area; red pixels: nonsuitable area. The map size is representative of the modeling boundaries for each species. (a) *Abatus cordatus*, (b) *Brisaster antarcticus*, (c) *Ctenocidaris nutrix*, and (d) *Sterechinus diadema*

For each species, SDM were produced with past [1955–1974] and future environmental predictions [2050–2099] to assess the magnitude of species distribution range shift with changing environments between periods (Figures 4 and 5; Table 2). For all species, distribution range shifts are relatively weak between [1955–1974] and [2005–2012], with *E*_instability_ being included between 0.02 ± 0.01 (for *S. diadema*) and 0.10 ± 0.04 (for *B. antarcticus*) (Table 2). Projections for [1955–1974] indicate smaller distribution range compared to the present‐day conditions, *A. cordatus* excepted. The distribution of *B. antarcticus* is restricted to the central and eastern parts of the Kerguelen Plateau (Figure 4). High *E*_overlap_ values (0.44 ± 0.03), however, indicate that the most suitable areas have not markedly changed between [1955–1974] and [2005–2012]. For [1955–1974], *C. nutrix* is predicted on the northern Kerguelen Plateau and other sub‐Antarctic archipelagoes (Figure 4). Comparison between [1955–1974] and [2005–2012] indicates an increase in habitat suitability in [2005–2012] with a poleward shift of suitable environments and more suitable areas in South Georgia and in the northern part of the Antarctic Peninsula (Figure 4). For *S. diadema*, suitable areas are also predicted to increase in the southern part of the Kerguelen Plateau and near the western Antarctic Peninsula region in [2005–2012] compared to [1955–1974] (Figure 4).

**Table 2:** Comparison of model outputs using the metrics developed by Crase et al. (2015); see equation details in Table 1. Values correspond to mean and standard deviation of 100 model replicates. Suitable area defined as the number of pixels for which species distribution probabilities are higher than the MaxSSS threshold value

	Suitable area			*E*_occupied_			*E*_instability_		*E*_overlap_	
	[1955–1974]	[2005–2012]	[2050–2099]	[1955–1974]	[2005–2012]	[2050–2099]	[2005–2012] vs [1955–1974]	[2005–2012] vs [2050–2099]	[2005–2012] vs [1955–1974]	[2005–2012] vs [2050–2099]
*Abatus cordatus*	218 ± 143	212 ± 121	53 ± 161	762.7 ± 28.4	731.6 ± 18.1	732.1 ± 107.2	0.09 ± 0.02	0.23 ± 0.09	0.32 ± 0.02	0.28 ± 0.04
*Brisaster antarcticus*	1,188 ± 604	1,522 ± 426	4,184 ± 722	1,888.1 ± 132.4	1,956.6 ± 40.9	2,711.7 ± 268.1	0.10 ± 0.04	0.42 ± 0.12	0.44 ± 0.03	0.61 ± 0.06
*Ctenocidaris nutrix*	3,924 ± 2,195	4,550 ± 2,259	6,408 ± 4,933	10,090.2 ± 242.1	10,296.6 ± 274.8	11,395.9 ± 1,009.8	0.04 ± 0.01	0.26 ± 0.07	0.42 ± 0.01	0.45 ± 0.04
*Sterechinus diadema*	9,559 ± 3,187	9,813 ± 3,007	8,899 ± 1,648	13,054.2 ± 228.4	13,427.6 ± 222.6	13,895.4 ± 631.4	0.02 ± 0.01	0.12 ± 0.02	0.44 ± 0.01	0.45 ± 0.02

The predicted distribution shifts are much more marked between [2005–2012] and [2050–2099] than between [1955–1974] and [2005–2012], with *E*_instability_ values being included between 0.12 ± 0.02 and 0.42 ± 0.12. However, high *E*_overlap_ values between [1955–1974] and [2050–2099] show that suitable areas are mostly conserved despite distribution shifts, *B. antarcticus* excepted (+175% increase in suitable area between [2005–2012] and [2050–2099]). *A. cordatus* is the species with the lowest *E*_overlap_ value between [2005–2012] and [2050–2099] despite minor differences in *E*_occupied_ values between these two periods. This weak overlap indicates that future distribution of *A. cordatus* is predicted to shrink in [2050–2099] around the Kerguelen Islands (Figure 5). This marked reduction is in line with the high contribution of seafloor temperature amplitude to the model (32.9%), the preference of *A. cordatus* for high amplitudes (Figure 2e; Figure S2), and conversely, the predicted decrease in these values for [2050–2099] (Figure S1). *B. antarcticus* shows the highest *E*_instability_ (0.42 ± 0.12) and *E*_overlap_ (0.61 ± 0.06) values, which predict a high stability of suitable areas in [2005–2012] and their expansion in [2050–2099] over the entire Kerguelen Plateau (Figure 5). This is in line with predictions of higher and more widespread chlorophyll a concentrations over the Kerguelen Plateau according to scenario RCP 8.5 (Figure S1). For *C. nutrix* and *S. diadema*, the high overlap values (between 0.42 ± 0.01 and 0.45 ± 0.04, respectively) indicate minor changes in the extent of suitable areas, but binary maps (Figure 5) indicate noticeable distribution shifts. The distribution of *C. nutrix* is predicted to shift poleward, with a decrease in suitable areas on the northern Kerguelen Plateau and in lower latitudes (<−50°S), and an increase along the western Antarctic Peninsula and in the eastern Weddell Sea (Figure 5). The future distribution of *S. diadema* is also predicted to decrease in the lower latitudes, with a poleward shift of preferential areas (Figure 5), in coastal waters of the northwestern Antarctic Peninsula in particular, but suitability is also predicted to decrease in the southernmost areas of the Peninsula (Figure 5).

### 3.5 Environmental shifts and evolution of ecological niche space

**Figure 6:**
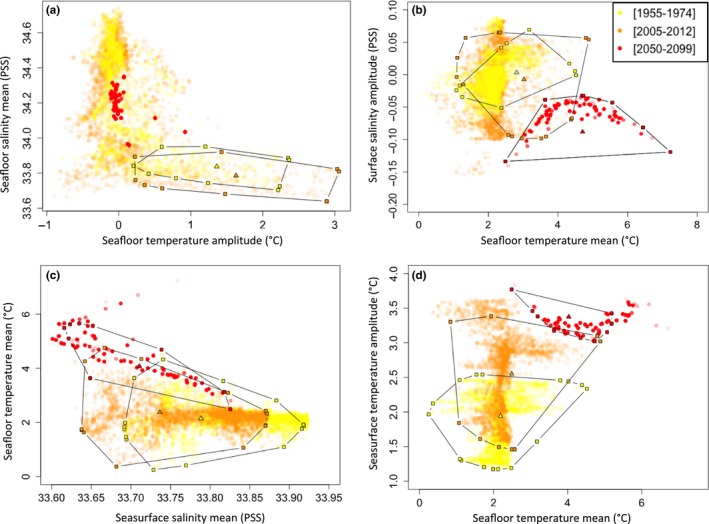
Occupied environmental subspaces modeled for [1955–1974], [2005–2012], and [2050–2099] for (a) *Abatus cordatus*, (b) *Brisaster antarcticus*, (c) *Ctenocidaris nutrix*, and (d) *Sterechinus diadema*. Environmental spaces were defined by predictors that most contribute to the respective SDM (Figure 2e). Background dots: environmental values present on the Kerguelen Plateau. Convex hulls delineate the environmental subspace preferentially occupied by species (environmental values of pixels for which values are higher than the MaxSSS value). Triangles: centroid position of the convex hulls. Colors correspond to the different time periods

To compare the size of species realized niches between the three periods (Figure 6), the environmental subspace occupied by each species was delineated by plotting the values of environmental descriptors that contribute the most to the models (Figure 2a'–d'). *A. cordatus* is the species that differs the most from the three other echinoids, with a niche space restricted to a narrow range of seafloor temperature amplitudes and mean salinity values (between 33.6 and 33.9 PSU) (Figure 6a). Projections for [2050–2099] indicate unsuitable conditions for the species on the Kerguelen Plateau (Figure S1), with values of seafloor temperature amplitudes and mean salinity values plotted outside of the species niche (Figure 6a), suggesting local potential extinction. In contrast, the niche space occupied by other species almost extends to the total environmental conditions available, suggesting wide fundamental niches (Figure 6b–d).

## 4 DISCUSSION

### 4.1 SDM limitations and perspectives

Modeling species distribution in the Southern Ocean is challenging. The paucity of available data is a major limitation to analyses that are restricted to presence‐only data models, usually considered less reliable and less efficient than presence–absence or abundance data models (Brotons et al., 2004). In addition, presence‐only datasets can be heterogeneous in space and time (compilation of 150 years of sampling in the present case study), which can influence modeling performances (Guillaumot et al., in press; Newbold, 2010; Tessarolo et al., 2014). SDM performed with spatially biased presence‐only data must consider these limitations and apply appropriate algorithms, protocols, and corrections (Barbet‐Massin et al., 2012; Guillaumot et al., in press; Phillips et al., 2009; Proosdij et al., 2016).

Strengthening sampling effort to characterize and model the entire distribution area of widely distributed species is one of the main priorities of Antarctic science, as recently pointed out by Kennicutt et al. (2014). Improving our knowledge of the Southern Ocean marine life also includes the development of efficient and robust modeling approaches. For instance, other environmental descriptors should be added to models in order to better characterize and depict species niches (Austin & Van Niel, 2011; Bradie & Leung, 2016). Integrating biotic interactions (Leach, Montgomery, & Reid, 2016) and combining correlative and mechanistic approaches (Briscoe, Kearney, Taylor, & Wintle, 2016; Buckley et al., 2010; Gutt et al., 2012; Morin & Thuiller, 2009) also constitute promising perspectives to improve our understanding of species distribution and potential response to changing environments.

Over the past decades, significant environmental changes were recorded on the Kerguelen Plateau, including a decrease in salinity and an increase in water temperatures between [1955–1974] and [2005–2012] (Frenot et al., 1993). According to IPCC scenario RCP 8.5 (IPCC Fifth Report, 2013), predicted changes for [2050–2099] imply an important water warming and freshening (Allan et al., 2013; Féral et al., 2016; Gutt et al., 2015). However, such predictions might be locally imprecise. The Kerguelen Plateau constitutes a major barrier to the flow of the Antarctic Circumpolar Current (ACC) (Park et al., 2014). Latitudinal heat flux and marine fronts' position are still debated, which could lead to different climatic scenarios (Vivier, Park, Sekma, & Le Sommer, 2015) and potentially different SDM for the region.

### 4.2 Model performance and species niche width

AUC values differ between SDM, with high and stable values for *A. cordatus* and lower scores for *B. antarcticus* and *S. diadema*. In previous works, Qiao, Soberón, and Peterson (2015) discussed the link between species niche width and the evaluation of SDM performances using true skill statistics and kappa indices. SDM produced for narrow‐niche species were proved to be associated with high sensitivity and high specificity scores. This is in line with the present results that show the highest AUC values for models performed for the narrow‐niche species, *A. cordatus*, and the lowest values for the wide‐niche species, *S. diadema*. When using AUC to evaluate the performance of models performed with presence‐only data, the maximum AUC value is given by the formula AUC = 1 − α/2, in which α corresponds to the fraction of the study area occupied by the species (Phillips, Anderson, & Schapire, 2006; Proosdij et al., 2016; Raes & ter Steege, 2007). Considering the extent of the species respective distribution areas, *A. cordatus* is the species with the smallest fraction of area coverage (α), which can account for the high AUC values of the model.

### 4.3 Major environmental drivers

Environmental descriptors that contribute the most to SDM vary between species according to the different ecological niches. For instance, the distribution of the deposit‐feeder *B. antarcticus* is strongly correlated with chlorophyll a concentrations and the species distribution is mainly predicted in regions with chlorophyll a blooms such as in the northeast of the Kerguelen Islands, in the vicinity of the Polar Front, and near the coasts of Crozet Islands (Park et al., 2014). In contrast, the nearshore species *A. cordatus* is mainly correlated with the values of seafloor temperature amplitudes, which are the highest in the shallow waters of the Kerguelen Islands.

The present results show the importance of using amplitude values in SDM, in association with other parameter metrics. They contribute to the SDM performed for the four echinoid species of this study as major descriptors. Bradie and Leung (2016) already discussed the importance of including descriptors of seasonal means and extremes in models. These descriptors were proved to further account for species distribution patterns than annual means, considering their stronger relationship with species niche width and ecological traits (i.e., growth and survival; see Franklin, 2009).

### 4.4 Species responses to environmental change

In the present work, we could generate robust models to assess the influence of changing environmental conditions on species distribution range, both in the geography and in the environment. The results confirm the sensitivity of species to changing environmental conditions. However, the modeled distribution maps and environmental spaces show diverging responses between species and time period. All species distributions were not proved to be significantly different between [1955–1974] and [2005–2012]. The occupied environmental spaces and suitable areas are quite similar both on the extent of the Southern Ocean and at the scale of the Kerguelen Plateau. Distribution range shifts are predicted to be much more important in the future, but the importance depends on (1) species niche width, (2) species distribution, (3) species sensitivity to seasonal variations, and (4) geographic features that may influence species distribution. Narrow‐niche, coastal, and endemic species such as *A. cordatus* should be the most affected by the predicted changes compared to wide‐niche echinoids such as *B. antarcticus*,* C. nutrix,* and *S. diadema*. The three last species are also predicted to show different distribution range shifts, from expansion (*B. antarcticus*) to poleward shift (*C. nutrix*) and latitudinal reduction (*S. diadema*).

Narrow‐niche species that are limited by environmental barriers, such as *A. cordatus*, cannot modify the occupied niche space while facing environmental changes. *A. cordatus* is endemic to coastal areas of the Kerguelen Plateau. It is a brooding species that is with no larval dispersal stage, which is a limiting factor to its dispersal capabilities and distribution range (David et al., 2005). Therefore, *A. cordatus* occupies the entire environmental space available around the Kerguelen Islands and cannot disperse to colonize other areas and find a climatic refuge. This corresponds to the “Wallace Dream” category as formalized by Saupe et al. (2012). In such a situation, the predicted changes in seafloor temperature amplitude and mean seafloor salinity are beyond the limits of the species niche, suggesting the potential extinction of *A. cordatus* in a near future.

The other studied species present a broader distribution range with no environmental barrier. Distributions are only limited by species environmental requirements, a situation formalized as the “Hutchinson Dream” category (Saupe et al., 2012). Species environmental suitability includes a large range of conditions, which encompass all the environmental conditions present on the Kerguelen Plateau. The distribution range of *B. antarcticus* is predicted to increase in the future, while the species suitable area remains constant due to the high chlorophyll‐a concentrations predicted in the Kerguelen and Crozet areas. Seafloor temperatures are predicted to increase according to scenario RCP 8.5, but they will remain within the range of the species niche and environmental suitability is not predicted to decrease. Despite a narrow distribution range in the present day, these results clearly suggest that the species has a large niche compared to *A. cordatus* and shows a higher potential resilience while facing predicted future changes.

The present distribution of *C. nutrix* extends over the entire Southern Ocean. However, the species preference for seafloor temperature is included between 1 and 4°C. Mean seafloor temperatures were predicted to increase between [1955–1974] and [2050–2099], leading to the progressive poleward shift of *C. nutrix*. In [1955–1974], the species was predicted to be mainly distributed in the northern part of the Kerguelen Plateau. According to future predictions, the distribution should be reduced in the northern Kerguelen Plateau and should reach the coasts of the western Antarctic Peninsula to the south, where temperature increase would match with the species preferences.

*Sterechinus diadema* is the Antarctic echinoid with the broadest distribution range. Between [1955–1974] and [2005–2012], suitable areas remain almost unchanged in both the sub‐Antarctic and Antarctic regions. In the future, southward distribution shifts are predicted on the Kerguelen Plateau due to the preference of *S. diadema* for water temperatures below 4°C. However, the species suitable area will not expand over the western Antarctic Peninsula due to the prevalence of sea ice and low chlorophyll a concentrations that limit the species distribution.

### 4.5 Effects of environmental change and conservation strategies

Water seafloor temperature and salinity were shown to have significantly varied since the 1950s, but major changes are still to come in a near future according to IPCC scenario RCP 8.5 (IPCC Fifth Report, 2013). Modifications in the seasonal amplitude of temperature and salinity variations should have decisive effects on costal marine ecosystems (CCAMLR 2008, 2013; Féral, Beurier et al., 2016; Gutt et al., 2018; Hureau, 2011; Sahade et al., 2015; Schram et al. 2016; Smale & Barnes, 2008). Predictions also suggest that other parameters of importance for marine life, such as chlorophyll a concentrations, should significantly vary in the future (Schram, Schoenrock, McClintock, Amsler, & Angus, 2015, 2016; Turner et al., 2013). Based on the available abiotic and biotic data, the present results suggest that the impact of these future changes on benthic organisms will have no equivalent in comparison with what happened in the past century. Results also illustrate that the response of benthic organisms may vary according to species‐specific physiology, life traits, biogeography, and ecological niche width, which is in line with previous works (Burrows et al., 2014; Clarke, Griffiths, Barnes, Meredith, & Grant, 2009; Peck, 2016; Peck, Morley, Richard, & Clark, 2014). Hence, benthic species of coastal areas might be particularly at risk, especially if they have narrow ecological niche and are endemic to the island. This stresses the need for implementing long‐term observing systems and monitoring the specific effects of environmental changes on coastal species.

The National Nature Reserve of the French Southern Territories contains areas in which fisheries activities are allowed and enhanced MPA devoted to conservation and research activities. The coastal areas of the Kerguelen Islands are now entirely included in this new enhanced MPA. The contribution of scientists has led to the implementation of the French Long‐Term Ecological Research PROTEKER observatory (IPEV program no. 1044, http://www.proteker.net, Féral, Saucède et al., 2016) for long‐term field observations of physical processes and ecosystem changes. In particular, the effects of temperature and salinity variations on coastal benthic species are monitored in the field (Féral, Saucède et al., 2016). The approach is coupled with ecophysiological experiments in the laboratory. Such a program is very complementary to the modeling approach presented in this study, as new field data should help improve the relevance of predictive models. Both approaches should provide scientific grounds to conservation managers of the French Southern Territories to develop efficient conservation plans in coastal areas of the Kerguelen archipelago.

## CONFLICT OF INTEREST

None declared.

## AUTHOR CONTRIBUTIONS

CG, AM, ME, and TS conceived the ideas and designed methodology; SFR provided a part of the data; and CG and TS wrote the manuscript and all the remaining authors contributed critically to the drafts and gave final approval for publication.
