# Supplementary material for: Benthic species of the Kerguelen Plateau show contrasting distribution shifts in response to environmental changes
Source: Ecol Evol. 2018 May 24;8(12):6210–25. doi: 10.1002/ece3.4091 (PMC6024116; doi:10.1002/ece3.4091)
Supplement: Supplementary file 1 [file ECE3-8-6210-s001.docx]

**Table S1:** List of environmental descriptors selected for the species distribution models. Asterisks ^(*)^ indicate that environmental layers are available for [1955-1974], [2005-2012] and future projections (RCP8.5, [2050-2099]). Spatial extent of the data: -78_-45°S/-180_180°W. Spatial resolution: 0.1°. Chlorophyll a, ice cover and POC data are not available for [1955-1974], they were considered similar to descriptors used for [2005-2012].

| Environmental descriptor | Unit | Description | Source for [1955-1974], [2005-2012] periods | Source for future projection RCP 8.5, [2050-2099] |
| --- | --- | --- | --- | --- |
| **Depth** | Meters | Bathymetric grid around the Kerguelen Plateau | This study. Derived from  [6] | - |
| **Sea surface temperature amplitude*** | °Celsius degrees | Difference between austral summer (mean January-March) and winter (mean July-September) sea surface temperature | World Ocean Circulation Experiment 2013 [1] | Derived from NOAA [5], ensemble of climatic models |
| **Seafloor mean temperature*** | °Celsius degrees | Mean seafloor temperature | This study. Derived from World Ocean Circulation Experiment 2013 [1] sea surface temperature layers | Derived from NOAA [5], ensemble of climatic models |
| **Seafloor temperature amplitude*** | °Celsius degrees | Difference between austral summer (mean January-March) and winter (mean July-September) seafloor temperature | This study. Derived from World Ocean Circulation Experiment 2013 [1] sea surface temperature layers | Derived from NOAA [5], ensemble of climatic models |
| **Sea surface mean salinity*** | PSS | Mean sea surface salinity | World Ocean Circulation Experiment 2013 [1] | Derived from NOAA [5], ensemble of climatic models |
| **Sea surface salinity amplitude*** | PSS | Difference between austral summer (mean January-March) and winter (mean July-September) sea surface salinity | World Ocean Circulation Experiment 2013 [1] | Derived from NOAA [5], ensemble of climatic models |
| **Seafloor mean salinity*** | PSS | Mean seafloor salinity | This study. Derived from World Ocean Circulation Experiment 2013 [1] seafloor salinity layers | Derived from NOAA [5], ensemble of climatic models |
| **Seafloor salinity amplitude*** | PSS | Difference between austral summer (mean January-March) and winter (mean July-September) seafloor salinity | This study. Derived from World Ocean Circulation Experiment 2013 [1] sea surface salinity layers | Derived from NOAA [5], ensemble of climatic models |
| **Mean surface chlorophyll a** | mg/m^3^ | Surface chlorophyll a concentration. Summer mean over 2002-2009 | MODIS AQUA (NASA) 2010 [2] | Derived from NOAA [5], ensemble of climatic models |
| **Sediments** | Categorical | Sediment features | [7], updated by Griffiths 2014 (unpublished) | - |
| Environmental descriptors | Unit | Description | Source for [1955-1974], [2005-2012] | Source for RCP 8.5,  [2050-2099] |
| **Geomorphology** | Categorical | Geomorphologic features | ATLAS ETOPO2 2014 [8] | - |
| **Slope** | Unitless | Bathymetric slope | [6] | - |
| **Mean seafloor oxygen concentration** | mL/L | Mean seafloor oxygen concentration over 1955-2012 | This study. Derived from World Ocean Circulation Experiment 2013 [1] sea surface oxygen concentration layers | Derived from NOAA [5], ensemble of climatic models |
| **Ice cover** | - | Proportion of time the ocean is covered by sea ice of concentration 85% of higher. Projection 2003-2010 | This study. Derived from Australian Antarctic Data Centre [3] | This study. Derived from Australian Antarctic Data Centre [3] |
| **POC export** | gC/m^2^/day | Particulate organic carbon 2002-2015 averages | This study. Published on Australian Antarctic Data Center [4] | Not available |

**References**

[1] WOCE 2013, link: <https://www.nodc.noaa.gov/OC5/woa13/woa13data.html> accessed 2016

[2] MODIS Aqua, link: <https://oceancolor.gsfc.nasa.gov/cgi> accessed 2016

[3] AADC, link: <http://webdav.data.aad.gov.au/data/environmental/derived/antarctic/> accessed 2017

[4] AADC POC export data, link: <https://data.aad.gov.au/metadata/records/Particulate_carbon_export_flux_layers>, created 2017

[5] NOAA, link: <https://www.esrl.noaa.gov/psd/ipcc/ocn/> accessed 2017

[6] Smith WH and Sandwell DT (1997) Global sea floor topography from satellite altimetry and ship depth soundings. Science. 277: 1956–1962.

[7] McCoy, F.W. 1991. Southern Ocean sediments: circum-Antarctic to 30°S. Marine Geological and Geophysical Atlas of the circum-Antarctic to 30°S. (ed. by D.E. Hayes) – Antarctic Research Series.

[8] Douglass LL, Turner J, Grantham HS, Kaiser S, Constable A and others (2014) A hierarchical classification of benthic biodiversity and assessment of protected areas in the Southern Ocean. PloS one. 9: e100551.

**Figure S1:** Layers of environmental descriptors on the extent of the Kerguelen Plateau (-46° to -56°S, +63° to +81°E). (A) Slope (°), (B) Chlorophyll-a concentration (mg.m^-3^), (C) surface mean salinity (PSS), (D) seafloor mean salinity (PSS), (E) Seafloor temperature amplitude (°C), (F) Seafloor mean temperature (°C). Numbers are related to time periods (1) [1955-1974], (2) [2005-2012], (3) RCP 8.5 scenario [2050-2099].

**Figure S2:** Species partial response plots showing range suitability (0-1). Blue lines correspond to LOESS regression curves and 95% confidence interval. (A1-4) *Abatus cordatus*, (B1-2) *Brisaster antarcticus*, (C1-2) *Ctenocidaris nutrix*, (D1) *Sterechinus diadema*.
